# Implementing physical activity programs for patients with cancer in current practice: patients’ experienced barriers and facilitators

**DOI:** 10.1007/s11764-019-00789-3

**Published:** 2019-07-25

**Authors:** Charlotte IJsbrandy, Rosella P. M. G. Hermens, Laura W. M. Boerboom, Winald R. Gerritsen, Wim H. van Harten, Petronella B. Ottevanger

**Affiliations:** 1 Radboud Institute for Health Science (RIHS), Scientific Institute for Quality of Healthcare (IQ healthcare), Radboud University Medical Centre Nijmegen, PO Box 9101, 6500 HB Nijmegen, The Netherlands; 2 Radboud Institute for Health Science (RIHS), Department of Medical Oncology, Radboud University Medical Centre Nijmegen, PO Box 9101, 6500 HB Nijmegen, The Netherlands; 3 Department of Radiation Oncology, Radboud University Medical Center, Nijmegen, The Netherlands; 4 Division of Psychosocial Research and Epidemiology, Netherlands Cancer Institute, Plesmanlaan 121, 1066 CX Amsterdam, The Netherlands; 5 Department of Health Technology and Services Research, MB-HTSR, University of Twente, PO Box 217, 7500 AE Enschede, The Netherlands

**Keywords:** Cancer survivors, Qualitative research, Healthcare quality improvement, Rehabilitation, Qualitative research, Health plan implementation

## Abstract

**Purpose:**

The present study aimed to identify patients’ experienced barriers and facilitators in implementing physical activity programs for patients with cancer.

**Methods:**

We interviewed 34 patients in focus-group-interviews from three different hospital-types. We included patients with cancer who were either receiving curative treatment or had recently completed it. Barriers and facilitators were explored in six domains: (1) physical activity programs, (2) patients, (3) healthcare professionals (HCPs), (4) social setting, (5) organization, and (6) law and governance.

**Results:**

We found 12 barriers and 1 facilitator that affect the implementation of physical activity programs. In the domain of physical activity programs, the barrier was physical activity programs not being tailored to the patient’s needs. In the domain of patients, lacking responsibility for one’s own health was a barrier. Knowledge and skills for physical activity programs and non-commitment of HCPs impeded implementation in the domain of HCPs. Barriers in the domain of organization included inconvenient place, time of day, and point in the health treatment schedule for offering the physical activity programs, inadequate capacity, inaccessibility of contact persons, lack of information about physical activity programs, non-involvement of the general practitioner in the cancer care process, and poor communication between secondary and primary HCPs. Insufficient insurance-coverage of physical activity programs was a barrier in the domain of law and governance. In the domain of physical activity programs, contact with peers facilitated implementation. We found no barriers or facilitators at the social setting.

**Conclusions:**

Factors affecting the implementation of physical activity programs occurred in various domains. Most of the barriers occurred in the domain of organization.

**Implications for Cancer survivors:**

An implementation strategy that deals with the barriers might improve the implementation of physical activity programs and quality of life of cancer survivors.

**Electronic supplementary material:**

The online version of this article (10.1007/s11764-019-00789-3) contains supplementary material, which is available to authorized users.

## Introduction

Physical activity (PA) has been shown to improve both the psychological and physiological functioning [1–5] of patients affected by cancer, by decreasing fatigue [2, 6–15] and improving cardiopulmonary fitness [6] and the quality of life [6, 9, 11, 16–21]. It also improves muscular strength [22], lean body mass, body fat levels [23], and self-esteem. Increasing evidence underlines the benefits of physical activity (PA) in preventing treatment side effects, improving progression-free survival, and increasing overall survival [4, 24]. Though maintaining a sufficient level of physical activity has been shown to counteract treatment side effects, multiple studies have shown that maintaining a physically active lifestyle during and after cancer is challenging for the patient [25, 26].

After cancer has been diagnosed, physical activity deteriorates distinctly. A low proportion of patients with cancer follow physical activity guidelines during treatment [27, 28], and it is well known that the physical activity levels of patients affected by cancer generally decline [29]. Additionally, even with extensive knowledge of the benefits of physical activity after cancer diagnosis, patients fail to return to pre-diagnosis activity levels after treatment [27, 28].

To improve the uptake of physical activity of patients with cancer, evidence-based guidelines recommend the implementation of physical activity programs or other initiatives to improve the uptake of physical activity during and after cancer treatment [6, 30–36]. However, it appears that the current uptake of physical activity programs is low [37–40] and widespread implementation is lacking [41]. A study found that only 17% of cancer treatment facilities offer a physical activity program [42]. Furthermore, there is scarce material on approaches to implement these guidelines on physical activity [43–46].

Like other new treatment approaches, perhaps physical activity programs need active implementation strategies that successfully deal with the barriers and facilitators involved [47]. Before these implementation strategies can be designed, further research is required to identify the barriers and facilitators affecting the implementation of physical activity programs during and after cancer treatment. This research can be compared to clinical practice, where a diagnosis is needed so that we can choose the right treatment [48]. The identification of these barriers and facilitators can be used to develop strategies for improved implementation of physical activity programs for patients. However, little is known about these barriers and facilitators influencing physical activity program implementation, so more exploration is needed. The present exploratory qualitative study therefore aimed to systematically identify barriers and facilitators affecting the implementation of physical activity programs among adult patients during and after the treatment of cancer.

## Method

We performed focus-group-interviews exploring factors that influence the implementation of physical activity programs among adult patients during and after the treatment of cancer. This qualitative study was carried out following the Consolidated criteria for Reporting Qualitative studies (COREQ) [49].

### Study population

The participants invited for the focus-group-interviews were recruited from three different hospitals in the Netherlands. As a sampling technique, we used purposeful sampling. We organized the focus-group-interviews in each participating hospital. Focus-group-interviews were performed until saturation was reached.

Ten to 12 adult patients with cancer were invited to participate in a focus-group-interview. Patients with cancer who were either receiving curative primary treatment or had recently completed it were eligible for interview. Their healthcare professionals (HCPs) asked if they were willing to participate when they were at the outpatient clinic and if so, their contact details were sent to one of the investigators. She contacted the patients by phone to tell them more about the study and to answer their questions. All the patients also received written information explaining the objectives and the process of the focus-group-interview and the investigator arranged an appointment for the focus-group-interview meeting. After the patients had been informed about the study and before the focus-group-interview started, they were asked for informed consent and permission to audiotape the focus-group-interview.

### Data collection

An interview guide was developed using theoretical models developed by Grol [50–52] and Flottorp [53] for identification of factors influencing implementation of care innovations.

Based on the theoretical models of Grol and Flottorp, the factors had been coded in the following six domains: (1) characteristics of the physical activity programs, (2) characteristics of the professionals, (3) characteristics of the patients, (4) characteristics of the social setting, (5) characteristics of the organization, and (6) characteristics of law and governance.

The physical activity programs can be guided by rehabilitation physicians, physical therapists, and sports-medicine physicians, who are specialized in physical activity during and after the treatment of cancer.

The focus-group-interviews were structured as follows: we asked the participants to describe their experience with physical activity programs. We explored obstacles or facilitators in detail as soon as they came up, for which we used the abovementioned theoretical models. The focus-group-interviews gave the participants a chance to talk freely, as well as to express their personal feelings about the obstacles to and facilitators of optimal care for their physical activity recovery. The investigators were not involved in any way in the care of the participants. The focus-group-interviews took about 90 min each and were conducted by two experienced investigators.

Before the focus-group-interviews began, we had collected patient characteristics by means of a short registration form. This form included questions about their age, sex, home setting, working conditions, cancer type, and types of therapy.

### Planned analytic approach and outcomes

Al interviews were audio-taped and afterwards literally typed up verbatim in manuscripts, using Microsoft Word. These manuscripts were imported in the qualitative software package Atlas.ti. We used version 7.6.16 for this purpose. The content analysis process, as described by Elo et al. [54] was used as methodology for the analysis. Two researchers qualitatively and independently coded the barriers and facilitators mentioned in the manuscript of the interviews. We coded the influencing factors in one of the six domains. Factors that had been identified, but had not been present in the domains, were added. The two investigators discussed their interpretation until consensus was reached.

## Results

### Patient characteristics

After analyzing the data of three focus-group-interviews, we concluded that saturation was sufficient. The participants invited for the focus-group-interviews were recruited from three different hospitals in the Netherlands: one university, one teaching, and one non-teaching. Table 1 outlines the characteristics of the participants. The three focus-group-interviews included a total of 34 adults with a history of cancer of the breast (38.2%), abdominal cavity (32.3%), pelvic cavity (8.8%), blood (14.7%), bone (2.9%), or lung (2.9%). Twenty-three of the 34 participants (68%) had finished their curative primary treatment and 11 participants were still receiving treatment at time of the interview. Of the participants, 88.2% had undergone surgery, 79.4% received chemotherapy, 47.0% radiotherapy and 17.6% hormonal therapy. Eleven participants treated in a university hospital took part in a focus-group-interview, as did 10 patients from a teaching hospital and 13 patients from a non-teaching hospital.

**Table 1:** Characteristics of the focus-group participants

Age (years)	Mean 60.4, SD (11.3), range (35–82)
Years since diagnosis	Mean 1.9, SD (0.2), range (0–5)
Years since treatment	Mean 1.7, SD (0.3), range (0–4)
	*n* (%)
Total amount of participants	34 (100)
Gender	
Male	12 (35.3)
Female	22 (64.7)
Nationality	
Dutch	33 (97.1)
Turkish	1 (2.9)
Household members	
Spouse and/or others	25 (73.5)
Alone	9 (26.5)
Partner	
Yes	24 (70.6)
No	10 (29.4)
Children	
Yes	23 (67.6)
No	11 (32.4)
Educational level	
Elementary	1 (2.9)
Intermediate	12 (35.3)
High	7 (20.6)
University/college	14 (41.2)
Work status	
Employed	20 (58.8)
Unemployed	14 (41.2)
Cancer type	
Breast	13 (38.2)
Abdominal cavity	11 (32.3)
Pelvic cavity	3 (8.8)
Hematological	5 (14.7)
Bone	1 (2.9)
Lung	1 (2.9)
Finished primary treatment	
Yes	23 (67.6)
No	11 (32.4)
Received treatments	
Surgery	160 (88.2)
Chemotherapy	43 (79.4)
Radiotherapy	69 (47.0)
Hormonal therapy	32 (17.6)
Symptoms during and after cancer treatment	
Total	24 (70.6)
Fatigue	11 (32.4)
Pain	3 (8.8)
Mental Problems	3 (8.8)
Lymph edema	1 (2.9)
Neuropathy	1 (2.9)
Nausea	1 (2.9)
None	10 (29.4)

The mean age of the focus-group participants was 60.4 years, with means of 1.9 years since diagnosis and 1.7 years since last treatment. Of the total amount of patients, 64.7% were female and 35.3% male. Ninety-seven percent had a Dutch background, one had a Turkish background. As highest level of education, 2.9% of patients has finished elementary education, 35.3% high school, 20.6% associated degree, and 41.2% university/college.

The participants came from all regions of the Netherlands. With regard to the social circumstances of the patients, 67.6% had children and 73.5% lived with their partner and/or children. Fifty-nine percent were employed at the time of the focus-group-interview. Seventy-one percent of the participants had symptoms or persistent side-effects from cancer and/or its treatment. The symptoms experienced were fatigue (32.4% of participants), pain (8.8%), mental problems (8.8%), lymph edema (2.9%), neuropathy (2.9%), and nausea (2.9%).

All the participants were aware of the existing physical activity programs for patients with cancer; 23.5% did not start any of these programs, 20.6% joined a physical activity program with strictly physical activity, 23.5% a physical activity program with psychological elements, and 32.4%, a psychologically oriented program that contained physical activity.

### Factors affecting the implementation of physical activity programs

The qualitative focus-group analysis revealed a variety of barriers and/or facilitators in the different domains outlined in Table 2. We found 12 barriers and 1 facilitator affecting the implementation of physical activity programs.

**Table 2:**
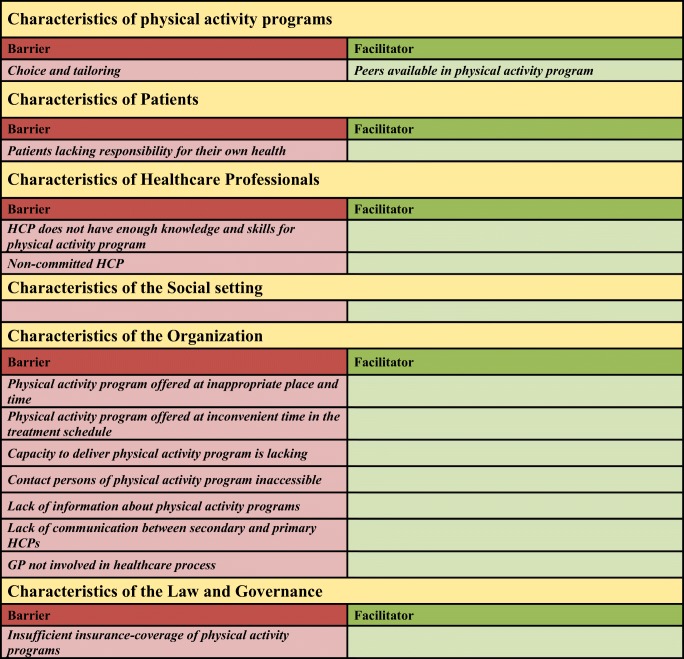
Factors affecting implementation of physical activity programs among adult patients with cancer

*GP general practitioner, HCP* healthcare professional

#### Domain: Physical activity programs

The key barrier in the domain of physical activity programs cited by the participants was that most physical activity programs contained fixed elements and were not tailored to the patients’ needs or capacities. Such programs were reasons to refuse joining a physical activity program, especially when all elements of the program were obligatory for participation. The forced set-up of a group or individual physical activity program was a reason to refuse. The participants preferred to have a choice of elements in the program and a choice of a group or individual physical activity program. They also preferred physical activity programs tailored to their individual needs so they could work on their own individual aspects that most needed improvement after cancer treatment.

The participants pointed out that contact with peers who had experience in dealing with cancer and its treatments was a facilitator that encouraged them to join a physical activity program. They felt more comfortable with people in the same circumstances. Often, sharing experiences was a reason to prefer peers. They also believed that involving peers ensured that the physical activity program was more suited to their needs as cancer survivors. Participants even preferred healthcare providers with their own experiences of cancer and its treatment.

Two quotes illustrating the barriers and facilitators in the domain of physical activity programs are:

“It would encourage me to join if the program were tailored to me as a person”

“I would be more receptive to advice or assistance if it would be provided by someone like me.”

#### Domain: Patients

In the patient domain, the lack of a sense of responsibility to participate was a main barrier to implementing physical activity programs. The participants believed that a responsible patient would ask for information, guidance, and referral. This would automatically encourage referral and participation in the physical activity program itself. They noted that a responsible patient would also take steps to fully participate into the physical activity program itself after referral, which would increase the participation percentage.

Two participants remarked about lacking responsibility for one’s own health:

“I think you should also ask for the program. You can’t have everything offered to you all the time. I think you also need to arrange some things yourself.”

“I asked my specialist for a physical activity program myself.”

#### Domain: Healthcare professionals

The focus-group participants explained that lack of knowledge and skills among HCPs resulted in a lack of qualified information for the patients. It also resulted in a lack of guidance to find the right physical activity program and a referral for joining the physical activity program. They also mentioned that HCPs who were not committed to physical activity programs could impede implementation. They did not always receive support from their HCPs for a successful referral, even if the patients themselves were aware of the need to participate in a physical activity program.These quotes illustrate the barriers in the HCP domain:

*“*I was here in August for a check-up. Only the prostate-specific antigen was measured and no attention was given to my status of physical activity.”

“I think they’ve never asked me.”

#### Domain: Social setting

We found no barriers in the domain of the social setting.

#### Domain: Organization

Multiple barriers in the domain of organization were noted, particularly the inappropriate place, time of day, and point in the health treatment schedule for offering a physical activity program. The participants named other barriers as well: inadequate capacity, inaccessibility of contact persons, and lack of information about physical activity programs, as well as non-involvement of the general practitioner (GP) in the cancer care and poor communication between hospital-based HCPs and primary care HCPs.

The participants reported that the accessibility of the physical activity program regarding place and time needed to be adjusted to their preferences. Travel distances or an inconvenient moment in time could hamper their participation. Some participants considered the hospital to be the best place for the physical activity programs during the treatment trajectory, since treatment and appointments could easily be combined with the time and place of the program. Other participants disagreed; they preferred to avoid the hospital after their therapy sessions. After the cancer treatment had been completed, most participants preferred to avoid the hospital. The physical activity programs should be easy to fit into daily life.

Some participants said that offering physical activity programs at inconvenient times in the treatment process would discourage them from participating. Many said that physical activity programs were offered during the treatment period when the participant did not feel ready, either physically or psychologically, to start rehabilitating. Others noted that physical activity programs were offered too late in the treatment process, and they felt that they had missed the right time in an earlier stage when they needed it.

Another point was the lack of capacity. Participants were confronted with waiting lists and a shortage of qualified personnel to mentor their physical activity programs. In addition, participants pointed out the lack of contact persons for information about physical activity programs, such as case managers in the hospital and the difficulty of reaching them. This could discourage them from trying to get information, guidance, and referral for the physical activity programs they needed. The participants said that the lack of information offered to them either verbally, on paper, via internet, or by other media was the main barrier in the organization domain.

In general, the communication and collaboration between the primary and secondary HCPs providing the physical activity programs was experienced as a barrier. The participants were aware of inefficient communication and collaboration about treatment, the state of the patient’s health, rehabilitation plan, and process. Participants also reported that, when they consulted their GPs with questions about a physical activity program, its benefits, or getting a referral, they felt that the GP was not involved in their cancer care process, lacked knowledge about physical activity programs, and/or lacked the skills and knowledge to advise and refer patients to physical activity programs. These shortcomings ultimately resulted in non-participation in physical activity programs. Other participants, who saw that their GPs were involved in the cancer care process, said that often the GP was the person who encouraged and helped them participate in physical activity programs. Representative quotes that capture these barriers are:

“The physical activity program was offered too late. I had already had a lot of problems and symptoms.”

“Isn’t there also a role for the general practitioner (GP)? I never heard anything from my GP. Whether I needed it was not questioned. I always had to contact him myself, and after a while I stopped doing that. After a while I thought... I approached him twice to tell... At least I call him, and a while later during the day he calls me back. I’m not satisfied with that. It should not be like that. If I did not contact him, I would probably never hear anything from him at all.”

#### Domain: Laws and governance

The focus-group-interviewees often named the barriers of insufficient insurance-coverage, lack of information, and uncertainty of insurance-coverage in the domain of laws and governance. For many participants, lack of insurance-coverage was a barrier to joining a physical activity program, since they did not have the money to pay for the physical activity program themselves.

Two quotes illustrating the insufficient insurance-coverage as a barrier are:

“With insufficient insurance-coverage, a physical activity program is not financially achievable for everybody.”

“I saw a physical therapist there. Then I asked if I could train with him. He said, ‘You have to pay for that yourself.’ Then I said, ‘Then it’s not possible’.”

## Discussion

This present exploratory qualitative study aimed to assess the barriers and facilitators influencing the implementation of physical activity programs among patients during and after cancer treatment. We found 12 barriers, most of which in the domain of the organization and one facilitator that affect the implementation of physical activity programs. The identified barriers are: programs that are not tailored to the patient’s needs; patients without a sense of responsibility regarding physical activity (programs); HCPs’ inadequate knowledge, skills, and commitment for physical activity programs; failure to ensure an appropriate place, time of day, and point in the health treatment schedule for offering the programs; lack of capacity; inaccessibility of contact persons; lack of information of physical activity programs; non-involvement of the GP in the cancer care process; lack of communication between primary and secondary HCPs; and insufficient insurance-coverage for physical activity programs.

### Domain characteristics of the physical activity programs

The characteristics of the physical activity programs are important for the success of implementation. The participants noted that physical activity programs that did not suit their needs discouraged them from joining the physical activity programs. Explorative studies have found that patients who survived cancer had various needs for physical activity and physical activity programs [55, 56]. Tailoring physical activity programs to individual needs has shown its effects in improving the physical activity outcome [57]. Tailored physical activity programs should be the first step toward successful implementation.

As do other studies, we found that bringing together peers might facilitate the implementation of physical activity programs by improving adherence to physical activity programs [58, 59]. Some even found this to be the most relevant enabler for patients to begin and continue these programs [60]. This positive influence is created by the feeling of belonging to a community (our physical activity group for patients with cancer) [60]. It also leads to the development of new networks, such as providing care for one another and carpooling. These networks increase social and practical support for joining the physical activity program.

### Domain characteristics of HCPs

In line with the findings of our study, recent studies have found that patients perceive that HCPs do not have enough knowledge and skills regarding physical activity programs [61]. We also found that any non-commitment or disinterest of HCPs toward the patient and their physical activity programs was experienced as a main barrier. Multiple studies report the increase of patients’ physical activity as a result of the commitment of HCPs to physical activity programs and their advice to patients to join such programs [50, 62–68]. In recent years, training programs for HCPs have been designed and used to overcome the knowledge gap for the HCPs. A natural result of extra knowledge might be commitment and interest, but extra attention to educate the HCPs about their important role in encouraging patients to increase their physical activity or to join a physical activity program might have additional beneficial effects.

### Domain characteristics of organization

In the domain of organization, patients pointed out that non-involvement of the GP in the cancer care process, inadequate communication between the GP and secondary HCPs, as well as a lack of communication between secondary and other primary HCPs, could form a barrier to implementing physical activity programs. In the Netherlands, cancer care is provided mainly by HCPs in secondary care. When cancer survivors transition from active cancer treatment to survivorship, the care is handed over to HCPs in primary care. The collaboration between primary and secondary care professionals must be optimal for successfully implementing physical activity programs. This is needed even more now that the number of cancer survivors is increasing. A shortage of survivorship care is expected, and multiple authorities have suggested increasing the role of primary HCPs in this area [69, 70]. The suggestion of optimizing the collaboration of primary and secondary HCPs in cancer care is not new [71, 72], but earlier studies have already shown the challenges of transitioning survivor care to primary HCPs because of the lack of communication with secondary HCPs [61]. It has been reported that patients’ perceptions of and trust in primary HCPs to understand the needs of the cancer survivors were low [61]. It would be helpful if the physical activity programs were implemented in a way that tackles the barriers in both primary and secondary care, so that a more team-based approach to survivorship care can be realized. To this end, an exploratory qualitative study aiming to identify the barriers and facilitators influencing the collaboration of the primary and secondary care for implementing physical activity programs would be welcome.

We found most of the barriers in the domain of organization. Implementation strategies directed toward improving the organizational aspects of the healthcare system itself probably require considerably more capacity and finance. Nonetheless, since the contextual factors of the organization seem to be important barriers to success, a strategy directed at the organization would greatly facilitate the implementation of physical activity programs. Evidence of the additional effects and cost-effectiveness of such a strategy in cancer care is still questionable and further exploration is needed.

### Domain characteristics of law and governance

Insufficient insurance-coverage of physical activity programs was a barrier in the domain of law and governance. The Dutch healthcare system is a market-oriented healthcare system [73]. The financing includes a mandatory universal basic health insurance that provides financial coverage of a comprehensive and uniform package of health services. Dutch residents can also obtain additional health insurance. Competing private insurers are responsible for negotiating contracts with healthcare provider agents for the patients who are insured for their services. This results in a large number and variety of insurance packages. The physical activity programs that rehabilitation physicians offer are financed mainly by the universal basic health insurance. The financial coverage of other physical activity programs depends on the patients’ specific health insurance package. Some argue that this healthcare system (implemented in 2006) fosters efficiency, enhances freedom of choice, and reinforces solidarity, while upholding the public values of accessibility, quality, and fiscal sustainability [74]. Despite this, the insurance system is controversial since it has been framed in a power conflict between health insurers and healthcare providers [73]. The prevailing opinion is that insurers are driven more by money than quality, with the patients caught in the middle [75]. The diversity of insurance packages and their coverage is enlarging the inequality of care, so that constant reform and improvement of the system to meet the needs of the patients is an inevitable future perspective [76]. Our focus-group interviewees homed in on the uncertainty of insurance-coverage and therefore the barrier of the uncertainty of the cost of joining a physical activity program. Our current study suggests that the controversial insurance problem probably also affects the implementation of physical activity programs. To overcome this barrier, the healthcare system needs political changes that can benefit implementing physical activity programs. Providing patients and their HCPs with clear information about insurance-coverage could be a reasonable beginning.

### Multifaceted implementation strategy

A multifaceted strategy focusing on different domains of barriers would be more successful in implementation in healthcare systems, because barriers often arise at different levels in a healthcare system [47, 50, 60, 77]. Recent studies in other patient groups show no consistent evidence for the effectiveness of multifaceted strategies (with, for example, combinations of communication, partnership, environmental strategies, social marketing, partnership with policy and environmental improvements) in increasing the “uptake of physical activity” in populations [48]. Nevertheless, we found barriers in almost all the domains we studied. Probably all the barriers affect implementation, and any one barrier affects the others. Therefore, designing and testing multifaceted strategies would be reasonable.

## Limitations

Some limitations of this exploratory study must be noted.

We conducted 3 focus-group-interviews with 10, 11, and 13 participants. Focus-group-interviews with more than 10 participants are difficult to control and they limit each person’s opportunity to share insights and observations. In addition, group dynamics change when participants want to, but are not able to describe their experiences. However, group sizes can have as many as 12–15 participants when there is a good moderator. We used an experienced moderator and experienced no problems concerning control and opportunities to share insights.

As a sampling technique, we used purposeful sampling. Even though convenience sampling (selection bias) could have occurred since motivated, more outspoken and easily accessible patients might be more willing to participate in the focus-group-interviews. To discourage this phenomenon, we stimulated healthcare professionals to ask all patients meeting the inclusion criteria for participation and organized the focus-group-interviews in the hospitals on the most convenient time agreed with the participants.

## Conclusion

This work contributes to a better understanding of barriers and facilitators that patients with cancer encounter in implementation of physical activity programs. Most barriers occur in the domain of the organization, such as the collaboration of the primary and secondary HCPs. Therefore, an exploratory qualitative study aiming to identify the barriers and facilitators influencing the collaboration of the primary and secondary HCPs to implement physical activity programs would be welcome. The results of this study can be used to develop a successful strategy for implementing physical activity programs and improving the quality of cancer care.

## Electronic supplementary material

ESM 1 (DOCX 25.8 kb)

## Abbreviations

COREQ: Consolidated criteria for reporting qualitative studies
HCP: Healthcare professional
GP: General practitioner
