# Supplementary material for: Implementing physical activity programs for patients with cancer in current practice: patients’ experienced barriers and facilitators
Source: J Cancer Surviv. 2019 Jul 25;13(5):703–12. doi: 10.1007/s11764-019-00789-3 (PMC6828940; doi:10.1007/s11764-019-00789-3)
Supplement: Supplementary file 1 — (DOCX 25.8 kb) [file 11764_2019_789_MOESM1_ESM.docx]

**Consolidated criteria for reporting qualitative studies (COREQ): 32-item checklist**

Developed from:

Tong A, Sainsbury P, Craig J. Consolidated criteria for reporting qualitative research (COREQ): a 32-item checklist for interviews and focus groups. International Journal for Quality in Health Care 2007. Volume 19, Number 6: pp. 349 –357

| **No** | **Item** | **Guide questions/description** |  |
| --- | --- | --- | --- |
| **Domain 1: Research team and reflexivity** | | |  |
| Personal Characteristics | |  |  |
| 1. | Interviewer/facilitator | Which author/s conducted the interview or focus group? | *Charlotte IJsbrandy*  *Rosella Hermens* |
| 2. | Credentials | What were the researcher's credentials? *E.g. PhD, MD* | *Charlotte IJsbrandy, MD*  *Rosella Hermens, PhD* |
| 3. | Occupation | What was their occupation at the time of the study? | *Charlotte IJsbrandy, MD and Researcher*  *Rosella Hermens, Associate Professor* |
| 4. | Gender | Was the researcher male or female? | *Both female* |
| 5. | Experience and training | What experience or training did the researcher have? | *Charlotte IJsbrandy participated in a course for qualitative research methods*  *Rosella Hermens has years of experience with focus group interviews* |
| Relationship with participants | |  |  |
| 6. | Relationship established | Was a relationship established prior to study commencement? | *No for both* |
| 7. | Participant knowledge of the interviewer | What did the participants know about the researcher? e*.g. personal goals, reasons for doing the research* | *All patients received orally and written information stating the objectives and the process of the focus group interview. Before the focus group began ,the patients were orally informed about the objectives, process of the focus group interview, process after the focus group interview, and both researcher’s goals and reasons for doing the research.* |
| 8. | Interviewer characteristics | What characteristics were reported about the interviewer/facilitator? e.g. *Bias, assumptions, reasons and interests in the research topic* | *The investigators were not involved in any way in the care of the participating patients.* |
| **Domain 2: study design** | | |  |
| Theoretical framework | |  |  |
| 9. | Methodological orientation and Theory | What methodological orientation was stated to underpin the study? *e.g. grounded theory, discourse analysis, ethnography, phenomenology, content analysis* | *Content analysis* |
| Participant selection | |  |  |
| 10. | Sampling | How were participants selected? *e.g. purposive, convenience, consecutive, snowball* | *10–12 adult cancer patients from three hospitals were invited to join the focus group. The patients, who were either receiving curative primary treatment or had recently finished it were asked to participate.* |
| 11. | Method of approach | How were participants approached? e*.g. face-to-face, telephone, mail, email* | *The patients were asked face to face for participation by their healthcare professionals when they were at the outpatient clinic.* |
| 12. | Sample size | How many participants were in the study? | *34* |
| 13. | Non-participation | How many people refused to participate or dropped out? Reasons? | *None of the patients who were willing to participate dropped out.* |
| Setting | |  |  |
| 14. | Setting of data collection | Where was the data collected? e*.g. home, clinic, workplace* | *Clinic* |
| 15. | Presence of non-participants | Was anyone else present besides the participants and researchers? | *No* |
| 16. | Description of sample | What are the important characteristics of the sample? *e.g. demographic data, date* | *Patients with cancer who were either receiving curative primary treatment or had recently finished it were asked to take part in the interview.* |
| Data collection | |  |  |
| 17. | Interview guide | Were questions, prompts, guides provided by the authors? Was it pilot tested? | *An interview guide was developed from Grol and Flottorp’s theoretical models for the identification of influencing factors.* |
| 18. | Repeat interviews | Were repeat interviews carried out? If yes, how many? | *Yes, three focus group interviews.* |
| 19. | Audio/visual recording | Did the research use audio or visual recording to collect the data? | *The interviews were audio recorded.* |
| 20. | Field notes | Were field notes made during and/or after the interview or focus group? | *If applicable, notes were made.* |
| 21. | Duration | What was the duration of the interviews or focus group? | *The focus group interviews took about 90 min each.* |
| 22. | Data saturation | Was data saturation discussed? | *Yes* |
| 23. | Transcripts returned | Were transcripts returned to participants for comment and/or correction? | *No* |
| **Domain 3: analysis and findings** | | |  |
| Data analysis | |  |  |
| 24. | Number of data coders | How many data coders coded the data? | *Two. Charlotte IJsbrandy, MD and Researcher*  *Laura Boerboom, MSc, Researcher* |
| 25. | Description of the coding tree | Did authors provide a description of the coding tree? | *The factors identified were classified within the earlier mentioned frameworks of Grol and Flottorp. We used the following domains: 1)* *characteristics of the physical activity programs, 2) characteristics of the professionals, 3) characteristics of the patients, 4) characteristics of the social setting, 5) characteristics of the organization and 6) characteristics of law and governance.* |
| 26. | Derivation of themes | Were themes identified in advance or derived from the data? | *The factors identified were classified in Grol and Flottorp’s frameworks. Factors identified but not already present in the model were added. The two investigators discussed their interpretation until consensus was reached.* |
| 27. | Software | What software, if applicable, was used to manage the data? | *Atlas.ti version 7.6.16.* |
| 28. | Participant checking | Did participants provide feedback on the findings? | *No* |
| Reporting | |  |  |
| 29. | Quotations presented | Were participant quotations presented to illustrate the themes / findings? Was each quotation identified? e*.g. participant number* | *Yes* |
| 30. | Data and findings consistent | Was there consistency between the data presented and the findings? | *Yes* |
| 31. | Clarity of major themes | Were major themes clearly presented in the findings? | *Yes* |
| 32. | Clarity of minor themes | Is there a description of diverse cases or discussion of minor themes? | *Yes* |
